# Establishment of a ccRCC patient-derived chick chorioallantoic membrane model for drug testing

**DOI:** 10.3389/fmed.2022.1003914

**Published:** 2022-10-06

**Authors:** Martine Charbonneau, Kelly Harper, Karine Brochu-Gaudreau, Alexis Perreault, Patrick P. McDonald, Nadia Ekindi-Ndongo, Claudio Jeldres, Claire M. Dubois

**Affiliations:** ^1^Department of Immunology and Cell Biology, Université de Sherbrooke, Sherbrooke, QC, Canada; ^2^Department of Medicine, Université de Sherbrooke, Sherbrooke, QC, Canada; ^3^Department of Pathology, Université de Sherbrooke, Sherbrooke, QC, Canada; ^4^Division of Urology, Department of Surgery, Université de Sherbrooke, Sherbrooke, QC, Canada

**Keywords:** ccRCC, chick chorioallantoic membrane, CAM assay, sunitinib, patient-derived xenograft (PDX), animal models, drug testing

## Abstract

Clear cell renal cell carcinoma (ccRCC) is an aggressive subtype of renal cell carcinoma accounting for the majority of deaths in kidney cancer patients. Advanced ccRCC has a high mortality rate as most patients progress and develop resistance to currently approved targeted therapies, highlighting the ongoing need for adequate drug testing models to develop novel therapies. Current animal models are expensive and time-consuming. In this study, we investigated the use of the chick chorioallantoic membrane (CAM), a rapid and cost-effective model, as a complementary drug testing model for ccRCC. Our results indicated that tumor samples from ccRCC patients can be successfully cultivated on the chick chorioallantoic membrane (CAM) within 7 days while retaining their histopathological characteristics. Furthermore, treatment of ccRCC xenografts with sunitinib, a tyrosine kinase inhibitor used for the treatment of metastatic RCC, allowed us to evaluate differential responses of individual patients. Our results indicate that the CAM model is a complementary *in vivo* model that allows for rapid and cost-effective evaluation of ccRCC patient response to drug therapy. Therefore, this model has the potential to become a useful platform for preclinical evaluation of new targeted therapies for the treatment of ccRCC.

## Introduction

Renal cell carcinoma (RCC), arising from renal tubular epithelial cells of the kidney, is one of the 10 most common cancers in adults (1, 2). There are three main subtypes of RCC, clear cell (ccRCC), papillary (pRCC) and chromophobe (chRCC), with ccRCC being the most aggressive and abundant histological subtype, accounting for 75% of RCCs and the majority of kidney cancer deaths (2, 3). Although localized ccRCC can be successfully treated by partially or completely removing the kidney, advanced ccRCC remains a clinical challenge with 5-year overall survival rates of 0–20% (4, 5). This high mortality is due to the fact that a significant number of patients do not experience disease stabilization or clinical benefits from currently approved targeted therapies, while most others will eventually progress and develop resistance to treatment (6–9). Therefore, there is an ongoing need to develop novel therapies or combinations of drugs that will more effectively treat ccRCC patients and for this, adequate drug testing models are essential.

There are various ccRCC models available for drug testing and each model system has unique advantages and disadvantages. *In vitro* cell line models are cost effective, with an ease of maintenance and manipulation and infinite replicative capacity that comply with the 3Rs, however they lack heterogeneity and contribution from the tumor microenvironment, making them a poor representation of patient tumors (6, 10). Organoids are an *in vitro* model with architecture more comparable to tumors from primary patient specimens (4). Organoids preserve tumor heterogeneity and therefore mimic original patient tissue better than cell lines so a number of studies are now investigating their use as a platform for drug screening (11–13). However, this model fails to recapitulate the tumor microenvironment and present many technical challenges and the need for standardization (4, 6). The zebrafish represents a simple *in vivo* model that has become widely used for cancer research due to their low cost and ease of genetic manipulation (14, 15). Recently, a VHL-mutant zebrafish model that could serve as model of early stage ccRCC and be used to study ccRCC development and tumor biology has been developed. However, this model is limited by the early mortality in zebrafish larvae and cannot be used to study more advanced ccRCC (16). Nonetheless, the most commonly used preclinical models for drug testing are mouse models that include cell-derived xenografts (CDX), patient-derived xenografts (PDX), or genetically engineered mouse models (GEMMs). GEMMs are immunocompetent models and thus can be used to study response to immunotherapies, but unlike most other models, they fail to fully represent the genetic and pathological phenotypes of human ccRCC (6, 17, 18). Mouse CDX models can recapitulate important aspects of the TME however, since they use cell lines, they also do not portray the heterogeneity of patients in the clinic (6). In contrast, PDX models most closely represent patient responses as they retain the histology and genetic signature of individual patient tumors and are therefore appropriate for elucidating mechanisms of response and resistance to candidate drugs (6, 19). Unfortunately, current mouse PDX models are very costly, time-consuming and require large numbers of mice, posing major ethical concerns (6, 20).

An interesting alternative PDX platform to the mouse PDX models is the chicken chorioallantoic membrane (CAM)-based PDX model (21). The chorioallantoic membrane is a highly vascularized extra-embryonic membrane connected to the developing embryo by an easily accessible circulatory system (22). The chick embryo is naturally immunodeficient, which allows for a high degree of success in the engraftment of multiple tissue types, including cancer cell line suspensions and patient-derived tumor explants, onto the CAM (23). Furthermore, ethical issues are mitigated in this model as the chick embryo is unable to perceive pain from the CAM tissue, which is not innervated (22). This model therefore meets the 3Rs principles for more humane research. Other distinct advantages of the CAM-based PDX model are that it is significantly less expensive than the mouse PDX model and can be used to test drug response of xenografts in a short time frame, an important feature for models used for personalized medicine (22, 24, 25). This model could therefore provide an interesting platform for preclinical characterization of novel therapies for ccRCC. In fact, previous studies have already determined that RCC tumor tissue can be engrafted on the CAM but the drug response of these tissues had not been tested (26–28).

In this study, we investigated the use of CAM as a complementary model for drug testing using ccRCC patient tumor tissues. Results indicated that tumor fragments from ccRCC patients successfully engraft on the CAM and retain their histopathological phenotype. Furthermore, treatment of the xenografts with sunitinib, an approved treatment for ccRCC, resulted in distinct responses from individual patients.

## Materials and methods

### Patient tumor specimen collection and tissue preparation

ccRCC patients' tumor tissue and corresponding adjacent healthy tissue were obtained from patients undergoing partial or radical nephrectomy at the Center hospitalier universitaire de Sherbrooke (CHUS) between the years 2018 and 2020. The study was conducted according to the guidelines of the Declaration of Helsinki under a protocol approved by the research ethics committee of the CHUS (#2017-1524). Written informed consent was obtained from all patients. Pathological diagnosis and grade were established by a urologic pathologist according to the WHO/ISUP 2016 grading system (29). Fresh tumor tissue specimens from the kidneys of patients were collected in the Department of Pathology of the Université de Sherbrooke and engraftment on CAM was performed within 2 h after resection. Necrotic tissues were carefully removed using a surgical blade prior to engraftment. A section of tumor tissue was also kept for histophathological analysis.

### ccRCC cell line

The Caki-1 cell line was obtained from the American Type Culture Collection. Cells were cultured in McCoy's 5A (Wisent, cat# 317-010-CL), supplemented with 10% FBS (Gibco, cat# 12483-020) and 40 μg/mL of gentamycin (Wisent, cat# 450-135-QL) in a humidified 95% air/5% CO_2_ incubator at 37°C.

### CAM-based PDX model establishment

Fertilized eggs from white leghorn chicken were obtained from the Public Health Agency of Canada (Nepean, ON) or the Couvoir Boire et Frères Inc. (Wickham, QC, Canada). The project was approved by the Ethics Committee of Animal Research of the Université de Sherbrooke (Protocol #054-17) and all experimental procedures involving chick embryos were conducted in accordance with regulations of the Canadian Council on Animal Care. CAM assays were performed as previously described (30) with the following modifications. Between embryonic day 8 and 10, depending on the time of surgery, freshly resected specimens were cut into tissue fragments with a diameter between 1 and 2 mm and implanted directly onto CAM in a mixture of Matrigel (VWR, cat# CACB354234) and EMEM culture medium (Wisent, cat# 320-005-CL) in a 1:1 ratio for a total volume of 20 μL. Caki-1 cell line suspensions (1 × 10^6^ cells/implant) were mixed with Matrigel in a 1:1 ratio in a total volume of 20 μL and implanted on CAM on day 9 of embryo development. For drug sensitivity assays, 2 days after the implantation of tumor fragments or Caki-1 cell lines, sunitinib (Sigma-Aldrich, cat# PZ0012) was injected in the CAM vasculature at concentrations indicated in figures. At day 16, chick embryos were euthanized. Vascularized tumor masses, without visible signs of necrosis, were considered to be successfully engrafted and were removed from CAM. Xenograft volumes were calculated using the formula (Dd^2^/2) and tissues were fixed in formalin for 24 h and embedded into paraffin for histopathological analysis. Drug treatments were considered effective when they resulted in at least 30% decrease (*p* < 0.05) in the volume of the lesions compared to the control xenograft group.

### Histology and immunostaining

Paraffin-embedded tissue sections from original and CAM tumors were freed of paraffin and rehydrated prior to staining. Harris hematoxylin (Fisher, cat#220-50-205) and Eosin (Fisher, cat# SH26500D) staining was performed for tissue morphology. Masson's Trichrome staining (ScyTek Laboratories Inc, cat# TRM-1-IFU) was used to detect collagen and performed following the manufacturer's instructions. Immunohistochemical staining was performed according to the standard avidin-biotin immunoperoxidase complex technique. The following antibodies were used: anti-Ki67 (Life technologies, MA5-14520) for proliferating cells, anti-vimentin (Calbiochem, cat#IF01-100UG), anti-CAIX (Santa Cruz, cat# sc-25599), anti-CK18 (Abcam, cat# AB93741) for ccRCC cells (31–33), anti-FAP (Abcam, cat# AB207178), and anti-αSMA (Agilent, cat# M0851) as markers of tumor stroma (34, 35), anti-CD105 (Abcam, cat# AB206419) a stem cell marker (36), or anti-cleaved-caspase 3 (NEB, cat# 9664S) a marker of apoptosis. Diaminobenzidine (Agilent, cat# K346711-2) was used for the detection of the labeled proteins and the sections were counterstained with Harris hematoxylin. Stained tissues were scanned at 40X magnification using the Hamamatsu NanoZoomer 2.0 RS slide scanner (Hamamatsu Photonics, Bridgewater, NJ, USA) for further analysis. Quantification of collagen positive or CK18-positive area was performed using ImageJ software (National Institutes of Health, Bethesda, MD, USA) as previously described.

### Statistical analysis

The GraphPad software (version 9.3.1) was used for statistical analysis. Significance was assessed by an unpaired Student's *t*-test (Mann-Whitney), or a one-way ANOVA (Kruskal-Wallis) as indicated in figure legends. A *p*-value smaller than 0.05 was considered significant.

## Results

### The CAM ccRCC PDX model allows proliferation of patient tumor fragments while maintaining their histopathological characteristics

To establish the CAM ccRCC PDX model, the *ex ovo* chicken embryo culture system was used to allow a broad access to the CAM vasculature for tumor implantation and drug treatments. For this study a total of 24 patients were recruited at the CHUS from January 2018 to March 2020. The Sherbrooke ccRCC patient cohort was established with the collaboration of the Urology Division and Department of Pathology at the CHUS. All patients gave informed consent, and their socio-demographic, medical record and follow-up information are presented in Table 1. Briefly, patients enrolled in the Sherbrooke RCC cohort had a median age of 67 years (range 45–86 years), with male patients accounting for 87.5% of the cohort. There was a prevalence of ISUP grades 2 and 3 (88% of patients at final diagnosis). Ninety-six percent of the tumors collected were primary, with one recurrence. To determine whether RCC tumor tissues can successfully engraft onto the CAM, tumor tissue from these patients was cut into small fragments (<5 mm^3^) and implanted onto CAM at day 9 of embryonic development. After 7 days, the embryos were euthanized to allow tumor extraction and further analysis. Overall engraftment rates were very high, with 100% per specimen and 94 % for individual implanted tumor fragments (Table 1).

**Table 1:** Clinical characteristics of the Sherbrooke RCC patient cohort.

**Clinical information**	**Number**	**%**
**Gender**		
Male	21	87.5
Female	3	12.5
**Median age**	67 years (45–86 years)	
**Diagnosis**		
Clear cell renal cell carcinoma		
ISUP Grade 1	1	4
ISUP Grade 2	11	42
ISUP Grade 3	10	46
ISUP Grade 4	2	8
**Tumor**		
Primary	23	96
Recurrence	1	4
**Tumor take rate**		
By specimen	24/24	100
By tumor fragment	757/807	94

**Figure 1:**
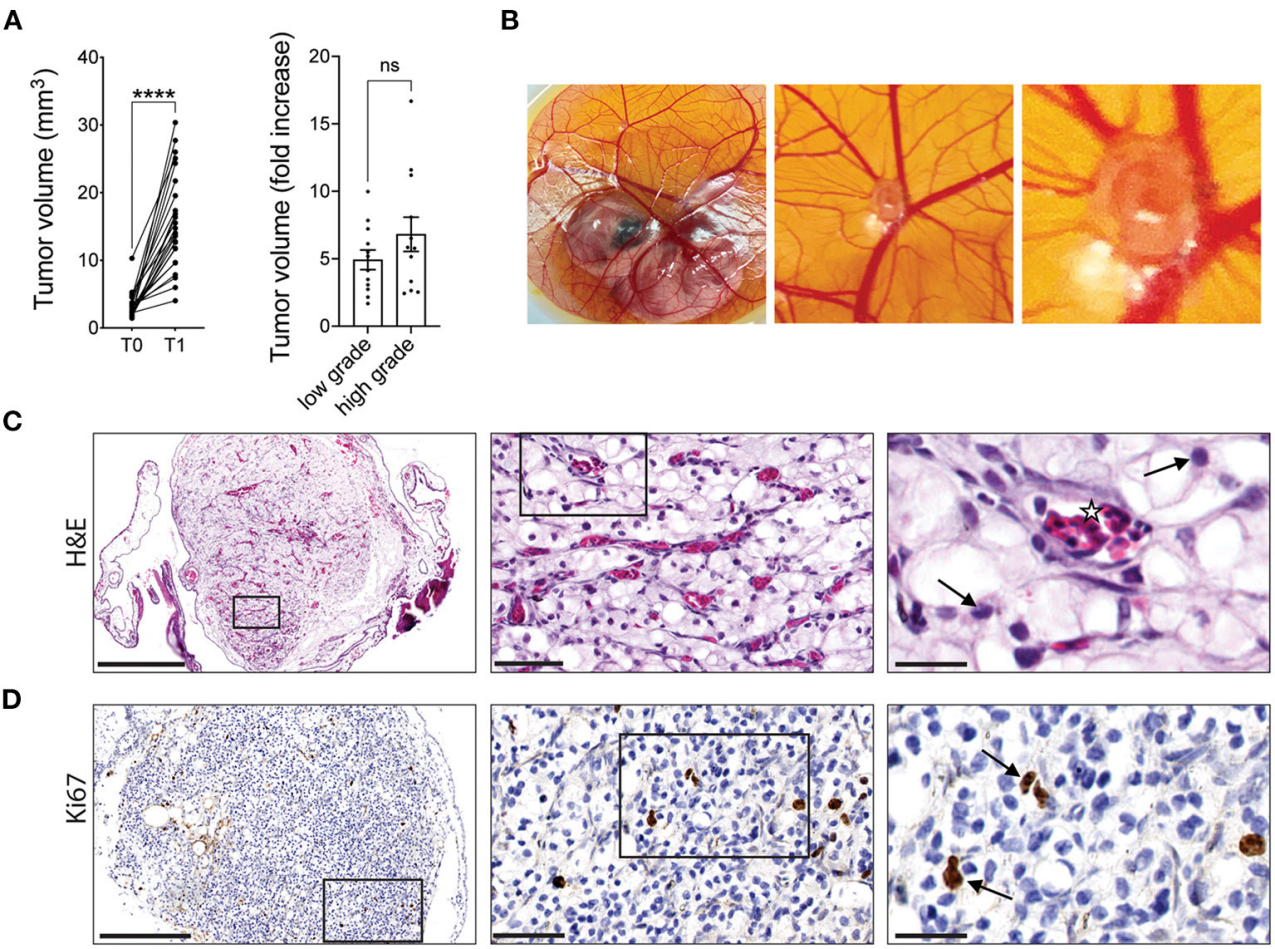
RCC tumor amplification and viability on CAMs. **(A)** Volume of original RCC tumor fragments (T0) and after one round of amplification on the CAM (T1; left graph). Fold increase in tumor volume from T0 to T1 for low grade (ISUP grade 1 and 2, 12 patients) or high grade (ISUP grade 3 and 4, 12 patients) tumors (right graph). Each dot represents the mean tumor volume of all implanted RCC fragments for an individual patient. **(B)** Representative image of a ccRCC tumor fragment grown on CAM with corresponding zooms. **(C)** H&E staining of a representative xenograft showing viable ccRCC nuclei (arrows) and capillaries filled with nucleated chicken erythrocytes (stars). **(D)** IHC staining of Ki67 in ccRCC CAM xenograft. Scale bars = 250, 50, and 25 μm. Values are expressed as the mean +/- SEM. Kruskal-Wallis test.

Seven days after implantation, xenografts were found to undergo significant expansion on the CAM with an average 5.8-fold increase in the mean volume of cultured tumors. Furthermore, the fold increase in tumor volume for high-grade tumors was greater than that for low-grade tumors (6.8 vs. 4.9-fold), however the difference was not statistically significant (Figure 1A). Gross observation of the tumor fragments growing on the CAM shows CAM vasculature feeding the xenograft (Figure 1B). The viability and vascularization of these xenografts were further evaluated by H&E and IHC staining. H&E staining showed that nuclear morphology was maintained after 7 days of cultivation on the CAM with no evidence of nuclear fragmentation, suggesting that the cells in the xenografts are viable. Capillaries filled with nucleated chicken erythrocytes (37) can be seen throughout the xenograft tissue, illustrating the ability of the CAM to irrigate the tumor xenografts (Figure 1C). Furthermore, staining for Ki67, a marker of cell proliferation, indicated the presence of cells within CAM tumor xenografts that are actively proliferating (Figure 1D). These results suggest that tumor xenografts from ccRCC patients are viable and can be expanded on the CAM.

**Figure 2:**
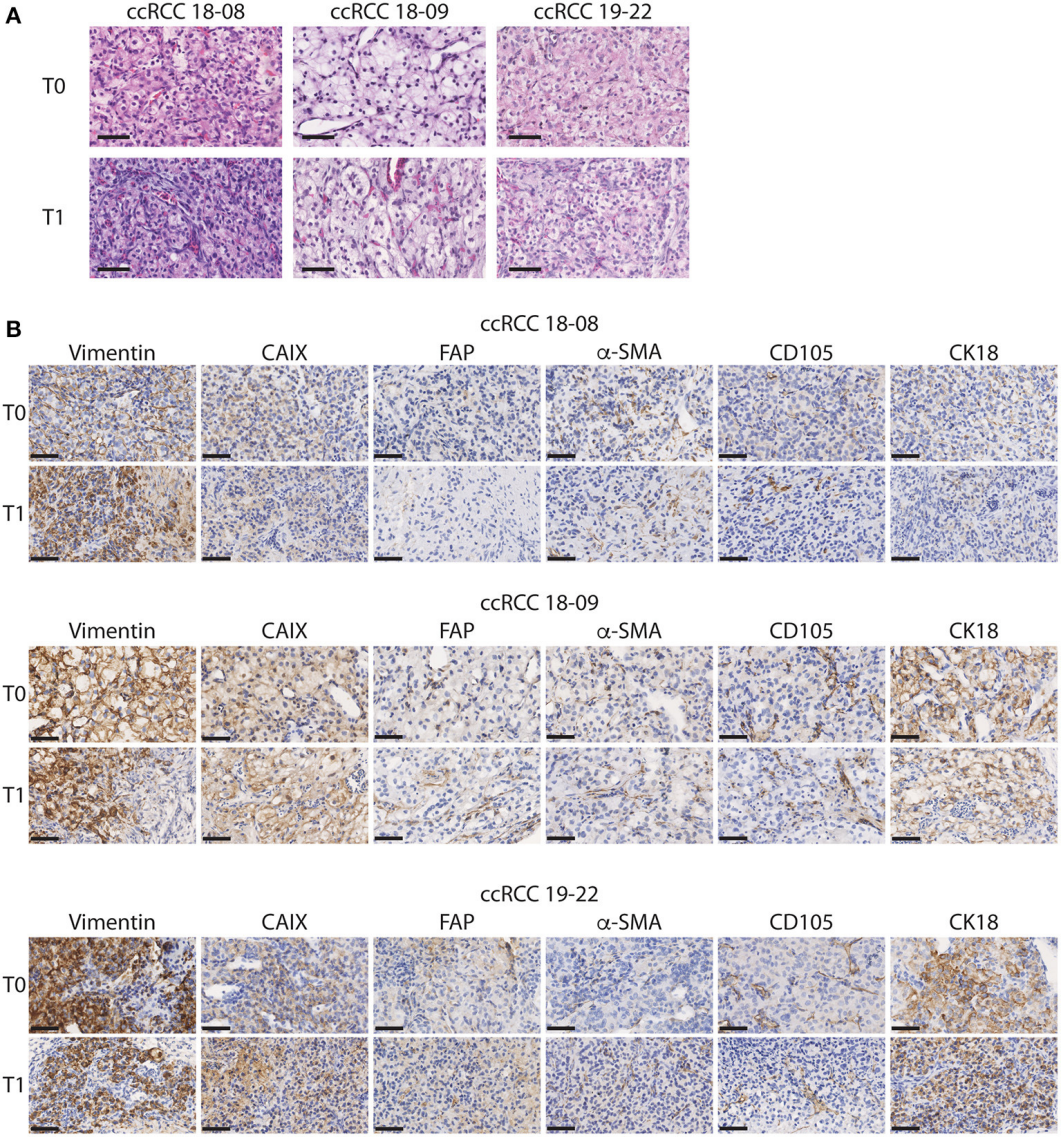
Histopathological characteristics of the original and xenograft ccRCC tumors. **(A,B)** Representative images of initial (T0) and xenografted tumor fragments expanded for 7 days onto CAM (T1) from 3 ccRCC patients. **(A)** H&E staining is shown. **(B)** Immunostaining for Vimentin, CAIX, FAP, α-SMA, CD105, and CK18 is shown for each patient. Scale bar = 50 μm, *N* = 5.

As a first approach to define whether CAM ccRCC xenografts recapitulate the characteristics of the original tumor, tissue sections from the original tumor (T0) and the tumor xenograft amplified on CAM (T1) were evaluated by H&E staining. In both T0 and T1 tissues, we observed the classic histological appearance of ccRCC that consists of a nested or alveolar growth pattern composed of cells with optically clear cytoplasm surrounded by a complex vascular network of capillaries (38) (Figure 2A). Next, the expression of various markers known to be expressed in ccRCC tumors was investigated by immunohistochemistry. These included cytokeratin 18 (CK18), an intermediate filament protein expressed in epithelial tissues and carcinomas, which is known to have a diffuse, strong staining pattern in ccRCC tumors (33), vimentin, a major constituent of intermediate filaments known to be highly expressed in ccRCC tumors (31), and CAIX, a hypoxia-responsive gene highly expressed in ccRCC tumors (39). Two microenvironment markers were also investigated including Fibroblast activation protein (FAP), expressed by cancer associated fibroblasts (CAFs), which are present at varying levels in ccRCC tumors (34), and α-SMA, a mesenchymal cell specific marker of myofibroblasts known to participate in ccRCC progression (35). Finally, we also investigated the stem cell marker, CD105 (endoglin), a coreceptor for TGF-*β* highly expressed in proliferating endothelial cells and a marker for cancer stem cells in ccRCC (36) (Figure 2B). During the *in vivo* expansion on CAMs, ccRCC tumors maintained a similar pattern and intensity of staining between original (T0) and xenografted (T1) tumors for all of these markers (Figure 2B). Overall, these results indicate that CAM tumor xenografts from ccRCC patients retain the histopathological characteristics of the original tumor including several features of the microenvironment.

### Patient-derived ccRCC xenografts display differential response to sunitinib

**Figure 3:**
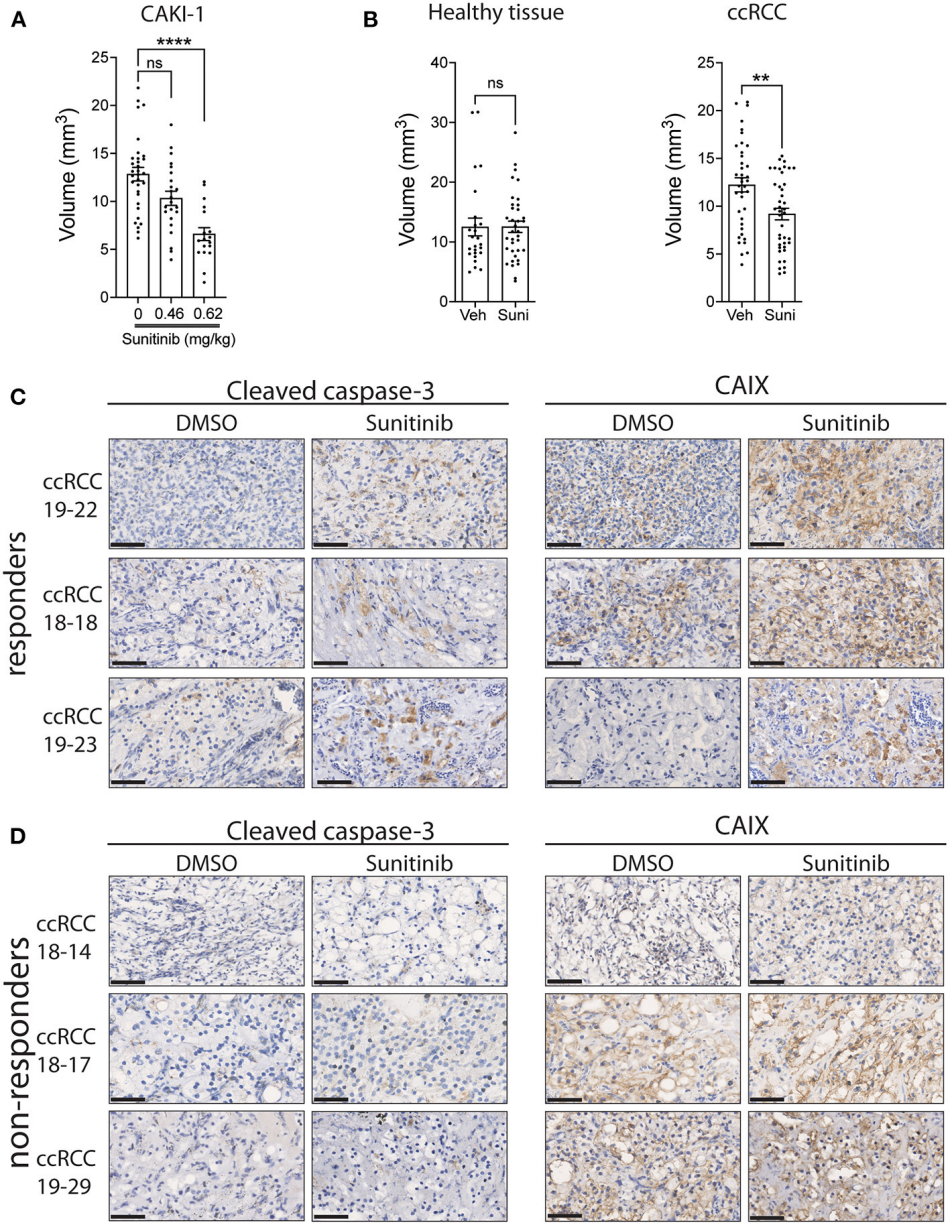
Effect of sunitinib treatment on xenografts grown on CAM. **(A)** Tumor volume of CAKI-1 cells grown onto CAM and treated with a dose-response of sunitinib (*N* = 5). **(B)** Fragments from renal parenchyma (healthy tissue; from three individuals) or ccRCC tissue (from three patients) were implanted onto CAM, treated with vehicle or sunitinib (0.62 mg/kg) at day 2 of implantation, and tumor volumes measured 5 days after treatment. **(C,D)** Representative images of CAIX and cleaved caspase-3 staining of xenograft tumors treated with vehicle (DMSO) or sunitinib in **(C)** 3 drug (sunitinib)-responder and **(D)** 3 non-responder CAM-ccRCC xenografts. Scale bar = 50 μm. Bars represent the mean +/- SEM. **p < 0,01, ****P < 0.0001, Mann-Whitney test.

We next evaluated the response of ccRCC xenografts to a commonly used drug for the treatment of ccRCC patients, sunitinib. Sunitinib is a multi-kinase inhibitor, targeting VEGFR1,2,3 and PDGFRα,β, which reduces angiogenesis, resulting in diminished oxygen supply to cancer cells, thereby inhibiting cancer cell growth (40). To evaluate the tolerance of the chicken embryo to sunitinib and to determine the optimal dose to be used in patient-derived xenografts, we first treated the sunitinib-sensitive Caki-1 ccRCC cancer cell line xenografts with sunitinib (41). Treatment efficacy was determined by measuring the volume of xenografts 5 days post-treatment compared to vehicle-treated xenografts. The effective dose of sunitinib was established by injection of 0.465 or 0.62 mg/kg of sunitinib into the vasculature of CAM implanted with Caki-1 cells. Results indicate that the 0.62 mg/kg dose, which is comparable to the 0.52–0.89 mg/kg dose normally administered to patients (42), significantly reduced tumor volume (Figure 3A). To confirm that the inhibitory effect of sunitinib is selective to tumor tissue, we administered 0.62 mg/kg sunitinib to CAM bearing healthy renal parenchymal tissue xenografts and found no significant effect on xenograft volume compared to the significant inhibition observed with ccRCC tumor xenografts (Figure 3B). We therefore used this optimal dosage in subsequent experiments.

To assess whether patient-derived ccRCC xenografts can display differential drug responses, tissue samples from 11 ccRCC patients were implanted on CAM and T1 xenografts treated with sunitinib or vehicle for 5 days. We observed a significant inhibition in xenograft volumes for 3 individual patients while 8 patients failed to respond to the treatment (Table 2). This 27% response rate was similar to the 12 to 36% clinical objective response rate reported for sunitinib treatment in the clinic (43–46). In an attempt to define whether the observed effects of sunitinib on ccRCC xenograft volume were due to its inhibitory actions on tumor growth and vascularization (40), sunitinib-treated xenografts were stained for caspase 3, a marker of apoptosis, and CAIX, a commonly used marker of hypoxia, which was found to be upregulated in response to anti-angiogenic agents (47, 48). The effect of sunitinib in drug-sensitive ccRCC xenografts was associated with increased levels of CAIX and cleaved caspase-3 immunostaining, whereas ccRCC xenografts that did not respond to sunitinib showed no changes in these markers. These results demonstrate the efficacy of the drug sunitinib to increase hypoxia and induce apoptosis in drug-sensitive xenografts (Figures 3C,D).

**Table 2:** Changes in patient-derived xenograft volumes following sunitinib treatment.

**Patient**	**Diagnosis**	**ISUP grade**	**% volume change**	***P*** **-value**
**18–14**	Clear cell renal cell carcinoma	2	−15, 61	NS
**18–17**	Clear cell renal cell carcinoma	3	−3, 05	NS
**18–18**	Clear cell renal cell carcinoma	3	**−63, 68**	**0.0106**
**19–22**	Clear cell renal cell carcinoma	3	**−40, 32**	**0.0036**
**19–23**	Clear cell renal cell carcinoma	2	**−39, 00**	**0.0073**
**19–24**	Clear cell renal cell carcinoma	3	−18, 48	NS
**19–26**	Clear cell renal cell carcinoma	3	10, 27	NS
**19–27**	Clear cell renal cell carcinoma	2	−13, 95	NS
**19-29**	Clear cell renal cell carcinoma	1	−8, 80	NS
**19–31**	Clear cell renal cell carcinoma	2	−7, 12	NS
**19–33**	Clear cell renal cell carcinoma	2–3	−23, 10	NS

Bold values: sunitinib-responder xenografts.

**Figure 4:**
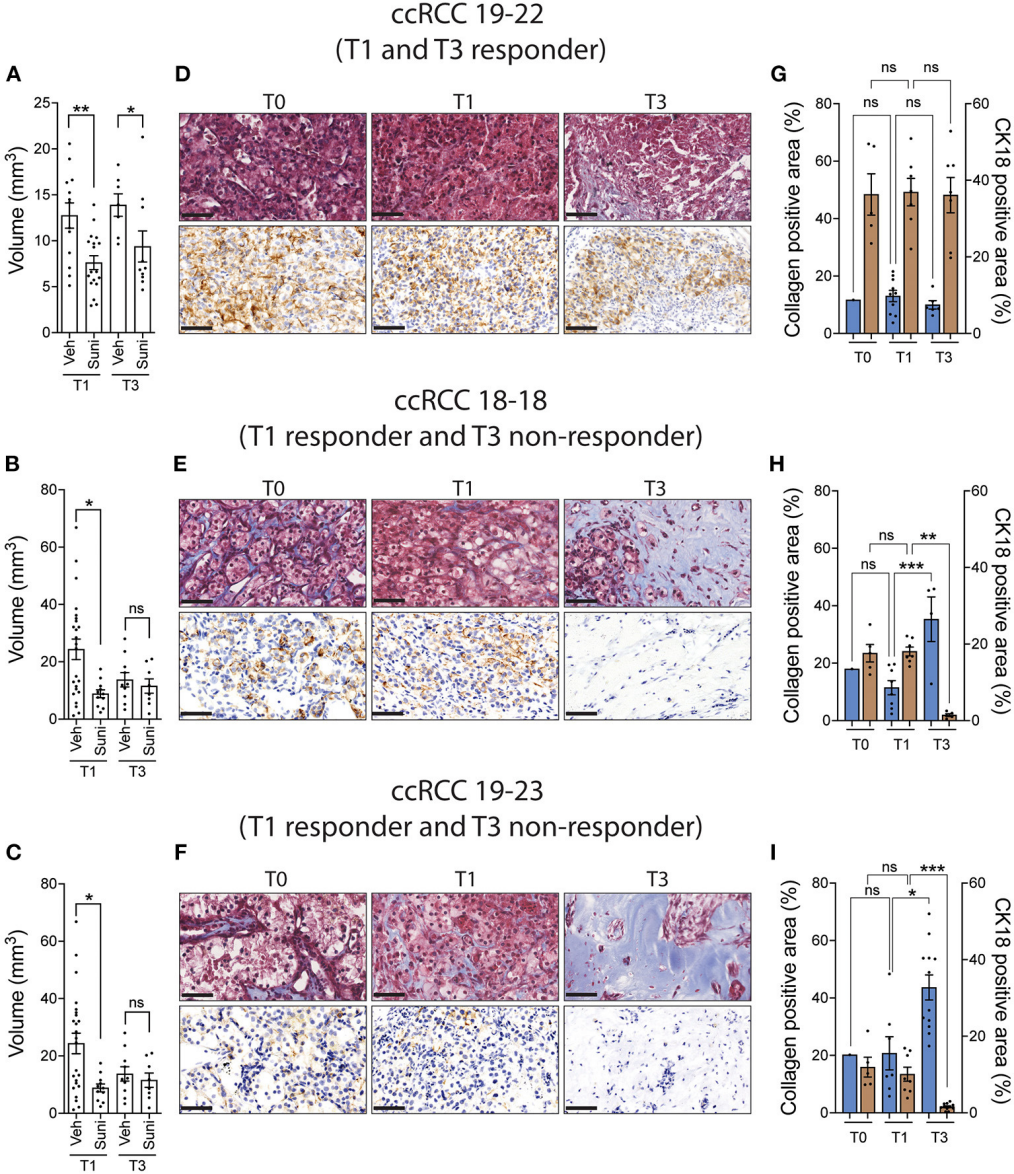
ccRCC xenograft response to sunitinib after multiple rounds of amplification on CAM. **(A–C)** CAM xenografts from the first (T1) and third (T3) rounds of amplification were treated with vehicle or sunitinib (0.62 mg/kg) and tumor volumes were measured 5 days after treatment. **(D–F)** Representative images of Masson's trichrome staining and CK18 staining in original tumor (T0) and T1 and T3 untreated xenografts (three patients). Scale bar = 50 μm. **(G–I)** Quantification of collagen positive area and CK18 positive area staining in original tumor (T0) and T1 and T3 untreated xenografts (three patients). Bars represent the mean +/- SEM. *p < 0.05; **p < 0.01, ***P < 0.001, Mann-Whitney test.

We then sought to determine whether the original tumor sample could be amplified for subsequent drug evaluation. To do this, ccRCC tumor fragments were implanted on CAM and serially passaged across multiple recipient eggs for up to three cycles of amplification. The results show that patient-derived tumor fragments that successfully engrafted onto CAM can be serially passaged onto multiple recipient eggs and continue to grow for up to 3 rounds of amplification (Supplementary Figure 1). Patient tumor xenografts were then treated with sunitinib at the first round (T1) and third round (T3) of amplification. The results indicate that the response to sunitinib observed at T1 was recapitulated in only one third of the PDX models tested at T3 (Figures 4A–C). Further analysis of T1 and T3 tissues by Masson's trichrome and CK18 staining revealed that CK18-positive ccRCC tumor cells were replaced by a collagen-rich tissue (blue staining) in T3 xenografts that ceased responding to sunitinib (T3 non-responder), while collagen levels remained similar between the original (T0) and T1 tissues (Figures 4D–I). These results validate that drug testing should be done on the first round of amplification of ccRCC tissue on the CAM.

## Discussion

The chick embryo model is increasingly being used as a complementary *in vivo* model for cancer research as it is a rapid, accessible, and cost-effective model that fulfills the 3R principles for more humane animal research. In this paper, we established a ccRCC PDX model using the chick chorioallantoic membrane (CAM) assay that allows cultivation of ccRCC patient tumor fragments in a 7-day time frame while maintaining their histopathological characteristics. In addition, this model allows the evaluation of the differential response of individual patients to sunitinib, a tyrosine kinase inhibitor (TKI) used for the treatment of ccRCC. Therefore, the CAM ccRCC model could be beneficial for rapid and cost-effective preclinical evaluation of drug therapies for the treatment of ccRCC.

In the present study, 100% (24/24) of patient tumors and 94% of individual tumor fragments were successfully implanted on the CAM resulting in an average 5.8-fold increase in the mean volume of cultured tumors. These results confirm that ccRCC patient tumor tissues can be grafted and expanded on the CAM, in agreement with results of previous publications (26–28). On the other hand, this tumor take rate is much higher than those found in mouse PDX models, which range from 15 to 30% for renal cell carcinomas (49–52). These differences might be explained by the fact that only tumor tissues from the most aggressive ISUP grades (3 and 4) could be successfully amplified in the PDX mouse models of ccRCC, with success rates of 45% for grade 4 and 18% for grade 3 (49). Such lack of successful implantation of low-grade ccRCC tissues can have consequences since the most common grades at diagnosis are 2 and 3, which correspond to 79–88% of ccRCC patients enrolled in the different cohorts (53, 54). Therefore, the majority of available ccRCC patient tissues would have poor tumor uptake in traditional mouse PDX models, whereas the CAM model offers an interesting advantage by allowing implantation of all grades of ccRCC patients.

Another advantage of the high tumor uptake rate of the ccRCC CAM model is the ability to test drugs directly during the first round of amplification on the CAM when tissue characteristics are most preserved. This differs from mouse models that require multiple passages of human tumor tissues in host mice to acquire sufficient tissue for drug testing, a process that can lead to changes in the composition of these xenografts (40). In fact, human-derived stromal components, including immune cells, endothelial cells, and fibroblasts, were found to be progressively replaced by murine stroma over time in several studies (55–59). The interaction of these tumor-associated stromal cells with cancer cells is known to play an important role in both tumor biology and drug responsiveness. Therefore, depletion of human stroma in mouse PDX models could affect the sensitivity of cancer cells to drugs (60–63). This is in line with our observation that the response to sunitinib treatment was lost after three passages in the CAM model, an event associated with a decrease in human ccRCC tissue as well as an increase in collagen-rich stroma.

In the ccRCC CAM model, xenografts were found to maintain the histopathological characteristics of their original tumor, including markers of ccRCC cells, stromal cells, and stem cells. Furthermore, the staining levels of these markers varied between individual patients, while remaining consistent between the original tumor and CAM xenografted tissue from the same patient. These results indicate that CAM PDX xenografts maintain the intertumoral heterogeneity of individual patients. This is important as maintaining patient tumor heterogeneity is one of the main advantages of PDX models that allows for better predictions of response to therapy compared to other preclinical models (40, 64).

The CAM model also has some limitations that must be taken into consideration. This model is limited by its short experimental window which consists of 7 days of tumor amplification. Therefore, the CAM model obviously cannot be used to study long-term drug effects, such as acquired resistance mechanisms, which normally take 20–30 days to develop in mouse models (65). Another major drawback of the CAM model is the lack of a fully functioning immune system, as chick embryos are not immunocompetent until day 18 of development, which limits testing of immunotherapies (66–68). Many ccRCC tumors are immunogenic containing a high number of immune cells and as a result targeted immunotherapies are becoming the treatment of choice for ccRCC (69, 70). Therefore, generating a humanized CAM model using autologous tumor and immune system would be an attractive approach to test these novel therapies. Humanized mouse models have already been successfully established by intravenous injection of live human PBMCs into immune deficient mouse models (40, 71–73), so development of a humanized-CAM model using a similar method should be feasible.

A critical aspect of evaluating any preclinical tumor model is its ability to reflect patient tumor drug response in the clinic. The percentage of ccRCC patients who responded to sunitinib in the CAM-PDX model (27%) was similar to the objective response rate (ORR) of ccRCC patients reported in the literature (12–35%) (43–46), indicating that the CAM ccRCC model is capable of responding to a commonly used drug to treat ccRCC patients. Unfortunately, we could not compare the patient tumor responses on the CAM to the individual patient response to therapy, as none of the patients in our cohort were treated with sunitinib. Future prospective studies with a cohort of ccRCC patients treated with chemotherapy would be needed to define whether the CAM-PDX model can be used to predict the response of ccRCC patients to drug therapy.

Overall, our results indicated that CAM-PDX model is a complementary *in vivo* model that allows for rapid and cost-effective evaluation of ccRCC patient response to drug therapy in the very tissue for which the drugs are being developed. As such, this model has the potential to become a useful platform for therapeutic decision making and cost-effective development of new targeted therapies.

## Data availability statement

The original contributions presented in the study are included in the article/Supplementary material, further inquiries can be directed to the corresponding author.

## Ethics statement

The studies involving human participants were reviewed and approved by Research Ethics Committee of the CHUS. The patients/participants provided their written informed consent to participate in this study. The animal study was reviewed and approved by Ethics Committee of Animal Research of the Université de Sherbrooke.

## Author contributions

CD, MC, KH, and PM: conception and design. MC, KH, AP, and KB-G: investigation. MC and KH: data analysis. KH: figure preparation and writing original draft. CD, MC, KH, KB-G, AP, NE-N, and CJ: review and/or editing. CD: supervision. CD, CJ, and PM: funding acquisition. CJ and NE-N: obtained the human tissue samples. All authors contributed to the article and approved the submitted version.

## Funding

This research was supported by grants from MEDTEQ/CQDM Innovation of Health, grant number 10-H Avatar and Fondation UdeS Merck Sharpe and Dohme. CD is a member of the FRQS-funded CRCHUS. KH, KB-G, and AP received an internship from Mitacs.

## Conflict of interest

The authors declare that the research was conducted in the absence of any commercial or financial relationships that could be construed as a potential conflict of interest.

## Publisher's note

All claims expressed in this article are solely those of the authors and do not necessarily represent those of their affiliated organizations, or those of the publisher, the editors and the reviewers. Any product that may be evaluated in this article, or claim that may be made by its manufacturer, is not guaranteed or endorsed by the publisher.
