# Supplementary material for: Establishment of a ccRCC patient-derived chick chorioallantoic membrane model for drug testing
Source: Front Med (Lausanne). 2022 Oct 6;9:1003914. doi: 10.3389/fmed.2022.1003914 (PMC9582329; doi:10.3389/fmed.2022.1003914)
Supplement: Supplementary file 1 [file Table_1.DOCX]

Supplementary Material

# Supplementary Figures

Supplementary Figure 1. ccRCC fragments (from 12 patients) were implanted on CAM for 3 rounds of amplification. Tumor volumes are shown. Values are expressed as the mean +/- SEM. **P<0.01, ***P<0.001, Kruskal-Wallis test.
